# Comparison of Outcomes in Obese Patients after Total Knee Arthroplasty with Neutral or Mild Varus: A Retrospective Study with 8‐Year Follow‐Up

**DOI:** 10.1111/os.14040

**Published:** 2024-03-31

**Authors:** Ya‐hao Lai, Wen‐xuan Zhao, Xiao‐yu Li, Ning Lv, Zong‐ke Zhou

**Affiliations:** ^1^ Department of Orthopaedic Surgery West China Hospital of Sichuan University Chengdu China; ^2^ Department of Pharmacy, State Key Laboratory of Biotherapy Sichuan University Chengdu China; ^3^ West China School of Public Health and West China Fourth Hospital, Sichuan University Chengdu China

**Keywords:** Alignment, Neutral, Obesity, Total Knee Arthroplasty, Varus

## Abstract

**Objectives:**

Residual varus after total knee arthroplasty (TKA) can affect functional outcomes, which may worsen in the presence of obesity. However, no studies were found to compare the outcomes of obese patients involving postoperative residual mild varus or neutral. The aim of this study was to compare postoperative complications and prosthesis survival, and functional outcomes for knees of obese patients with neutral or mild varus after TKA.

**Methods:**

We retrospectively reviewed 188 consecutive obese patients (body mass index ≥30 kg/m^2^) at our hospital who underwent TKA due to varus knee osteoarthritis from January 2010 to December 2015. The mechanical hip‐knee‐ankle axis angle was measured in all patients at admission and discharge. Knee functions were retrospectively assessed based on the Western Ontario and McMaster Universities Osteoarthritis Index (WOMAC) score, Knee Society Knee Score (KS‐KS), Knee Society Function Score (KS‐FS), Forgotten Joint Score (FJS), and range of motion (ROM). Continuous data were compared between knees with neutral or mild varus alignment using analysis of Student's *t* test or variance or the Kruskal–Wallis test as appropriate. For multiple comparisons of outcomes, we used Bonferroni–Dunn method to adjust *p*‐values. Categorical data were compared using the chi‐squared test.

**Results:**

Of the 156 knees in 137 obese patients who completed follow‐up for a mean of 8.32 ± 1.47 years, 97 knees were corrected from varus to neutral and 54 knees were kept in mild residual varus. Patients with mild varus knees had significantly WOMAC (8.25 ± 8.637 *vs.* 14.97 ± 14.193, *p* = 0.009) and better FJS (86.03 ± 15.607 *vs.* 70.22 ± 30.031, *p* = 0.002). The two types of knees did not differ significantly in KS‐KS, KS‐FS, or ROM. Although one patient with a neutral knee had to undergo revision surgery, there was no significant difference between two groups.

**Conclusions:**

For obese patients with osteoarthritis, preservation of residual varus alignment after TKA can improve functional outcomes without compromising prosthesis survival.

## Graphical abstract

Our study found that knees with mild varus showed significantly better joint functional scores in obese patients. For obese patients, preservation of residual varus alignment can improve functional outcomes without compromising prosthesis survival.

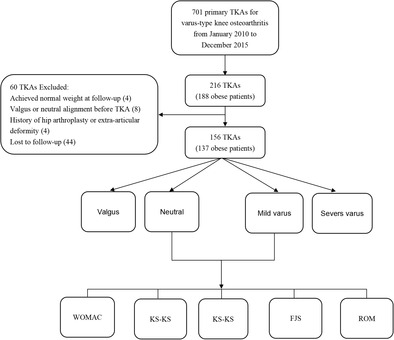

## Introduction

Since 1980, the prevalence of overweight and obesity has increased to the point that nearly a third of the world's population is overweight or obese.^1^ Obesity is a risk factor for osteoarthritis,^2^ so the proportion of patients undergoing total knee arthroplasty (TKA) who are obese is expected to increase.^3^ Obesity can lead to osteoarthritis that requires surgical treatment at an earlier age than in non‐obese individuals,^3^ and obesity can increase the risk of perioperative and long‐term complications.^4^ It may also increase risk of poor outcome and prosthesis failure after TKA.^5^

Some work has suggested that neutral mechanical alignment after TKA, defined as an adjustment within 3° of neutral, may prolong prosthesis survival and improve function.^6^, ^7^ However, other studies have failed to detect a significant difference in survivorship^8^, ^9^ or other outcomes^10^ after TKA in knees with neutral or mild varus alignment. Thus, further study is needed to clarify whether neutral alignment is beneficial for TKA patients. In particular, whether it benefits obese patients should be addressed, since neutral alignment is more difficult to achieve in these patients.^11^ In addition, obese patients may be at higher risk of aseptic tibial loosening regardless of coronal alignment,^12^ since the excessive body weight is transferred to surrounding bone.^13^

Therefore, whether there should be residual mild varus alignment after TKA, especially in obese patients, remains controversial. On one hand, there are a certain proportion of people with 3° or more varus as natural limb alignment in the physiologically normal population.^14^ More “physiological” prosthesis alignment may lead to better long‐term knee function. On the other hand, incomplete neutral alignment after surgery may prevent the mechanical bearing of the lower limb from passing through the center of the prosthesis, which may potentially accelerate the wear of the prosthesis, especially the polyethylene implant, and affect the survival time of the prosthesis in the long‐term results.^10^

Therefore, our study aimed to investigate whether: (i) postoperative neutral or mild varus alignment affected prosthetic survival in obese patients; (ii) whether neutral or mild varus alignment affected the joint function of obese patients.

## Methods and Materials

### Patients

Our study was designed in accordance with the Declaration of Helsinki and was approved by the Ethics Committee on Biomedical Research of West China Hospital of Sichuan University (approval 2012268), which waived the need for written informed consent. We retrospectively screened all patients at our hospital who underwent primary TKA for varus‐type knee osteoarthritis from January 2010 to December 2015. During this period, we performed 701 cemented TKAs in 663 patients, 188 of whom (216 knees) were obese, defined as a body mass index ≥30 kg/m^2^.^15^

**FIGURE 1:**
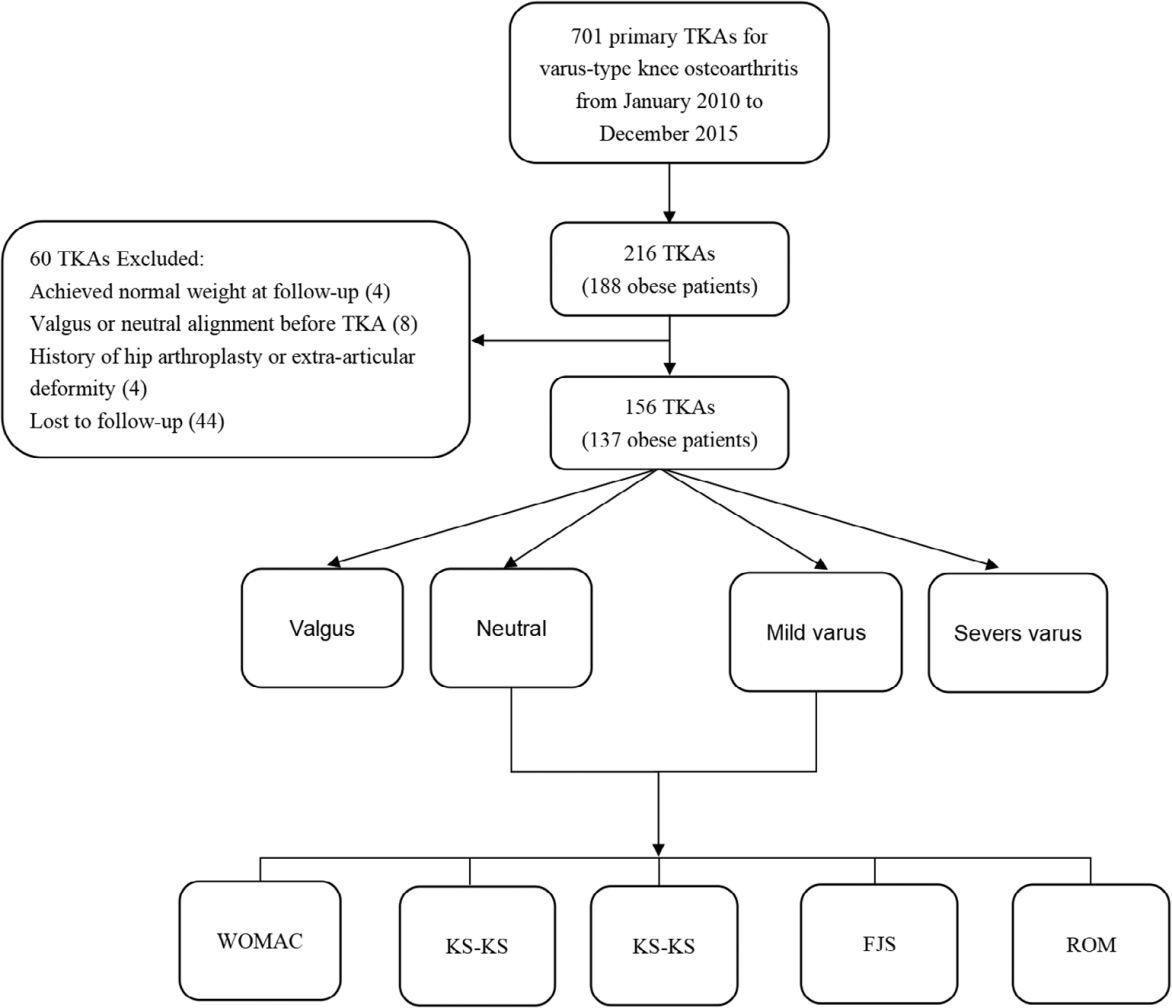
Flow chart.

The inclusion criteria were: (i) preoperative diagnosis of osteoarthritis; (ii) varus‐type knee before surgery; (iii) performing primary TKA; (iv) preoperative body mass index ≥30 kg/m^2^. Exclusion criteria were: (i) achieving normal weight at our follow‐up; (ii) valgus or neutral alignment before TKA; (iii) with a history of hip arthroplasty or extra‐articular deformity that could affect ipsilateral limb alignment; (iv) lost to follow‐up. In the end, we included 137 patients (156 knees) (Figure 1).

**FIGURE 2:**
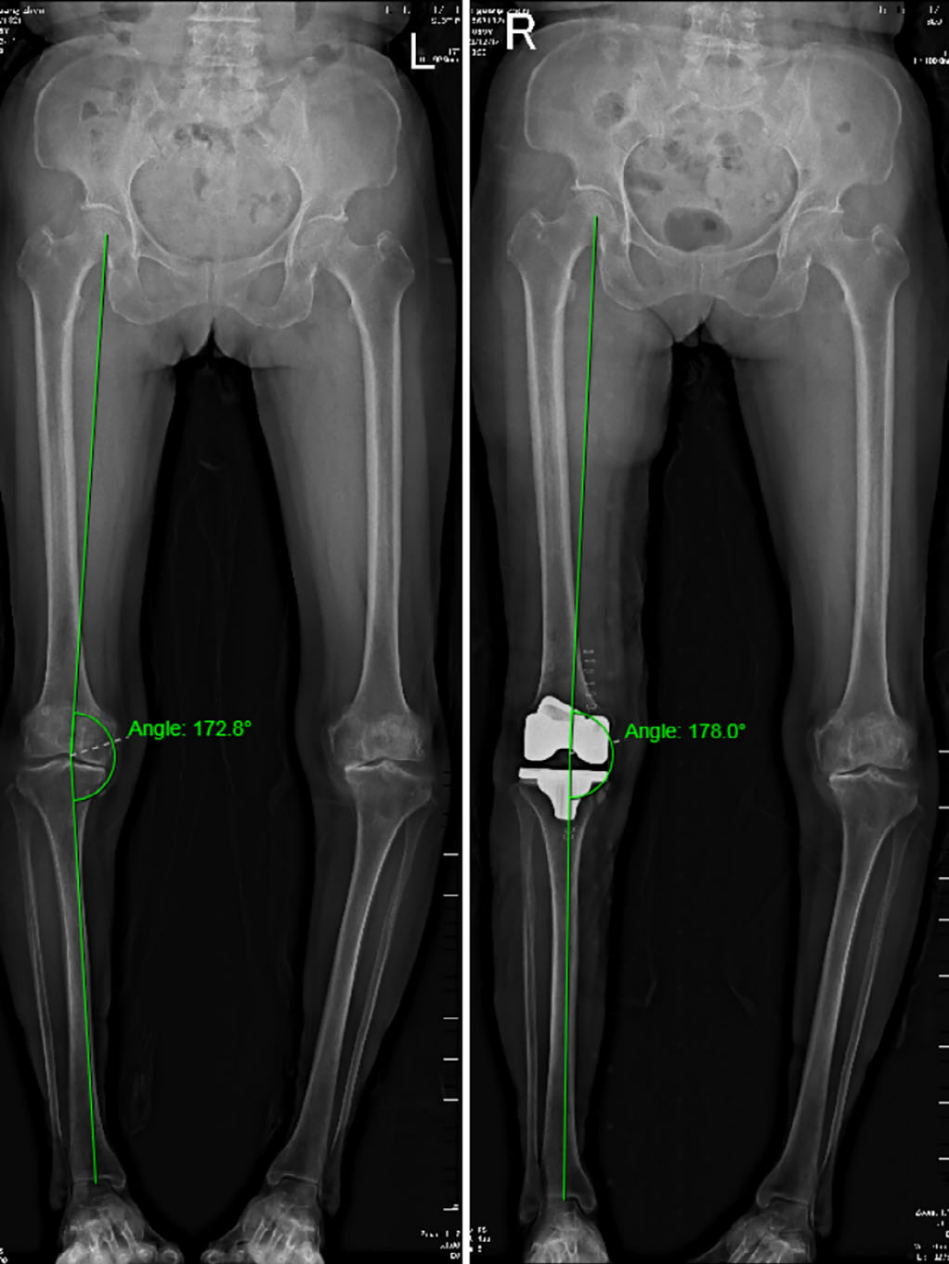
Method of calculation of HKA. The angle was measured on a standing, full‐length hip‐to‐ankle X‐ray. R, right. L, left. HKA, hip‐knee‐ankle angle.

The mechanical hip‐knee‐ankle axis angle (HKA) was defined as the angle between the femoral mechanical axis (the line between the midpoint of the femoral head and the midpoint of the femoral condyle) and the tibial mechanical axis (the line between the midpoint of the tibial plateau and the midpoint of the distal tibial articular surface)^16^ (Figure 2). HKA was measured in all patients at admission and discharge based on full‐length radiography conducted while patients were standing and the patella was oriented forward. Angles were determined independently by two investigators.

### Surgical Technique

All procedures were performed by one surgeon, who performed a midline skin incision and employed a medial parapatellar approach. After retracing the patella, the anterior cruciate ligaments and meniscus were completely resected. Osteotomy was performed using an intramedullary jig for the distal femur and extramedullary jig for the proximal tibia, and the valgus angle during osteotomy was determined according to preoperative full‐length radiographs to achieve neutral alignment.

On the femoral side, Whiteside's line and the anatomical transepicondylar axis were used as landmarks to position the intramedullary guides, while on the tibial side, the center of the tibial intercondylar eminence and the center of the ankle were used as landmarks to position the extramedullary guides. The tibia was cut, the posterior cruciate ligament was resected, and soft tissue was released according to the degree of preoperative flexion contraction. Depending on the patient's degree of varus, polyethylene spacers of varying thickness were inserted, or additional soft tissue was released. All patients received a Sigma fixed or rotating plant posterior‐stabilized total knee prosthesis (PFC, Johnson & Johnson/DePuy, Warsaw, IN, USA) and underwent patelloplasty without patellar resurfacing.

### Data Collection

Body mass index was calculated as weight (kg) divided by the square of the height (m). HKA was expressed as a deviation from 180°, with positive values indicating valgus and negative values indicating varus alignment. Two orthopaedic surgeons individually measured 50 randomly selected radiographs to assess the reliability of the measurements. The intraclass correlation coefficients for intra‐observer was 0.928 and 0.877 for inter‐observer reliability. Two orthopaedic surgeons simultaneously measured the HKA of the included cases and calculated the mean value. If the measurement error exceeded 1°, the measurement was repeated. Based on postoperative HKA, knee alignment was classified as valgus (HKA >3°), neutral (−3° ≤ HKA ≤3°), mild varus (−6° ≤ HKA < −3°), or severe varus (HKA < −6°). Most comparisons in this study were performed between knees showing neutral or mild varus alignment.

### Follow‐Up

Patients were followed up by outpatient visit. The patients were followed up at 1, 3, and 6 months after surgery, and then once a year. At last follow‐up, we measured range of motion (ROM), Western Ontario and McMaster Universities Osteoarthritis Index score (WOMAC), Keen Society Score (KSS), and Forgotten Joint Score (FJS). Prosthesis infection and aseptic prosthesis loosening were recorded to assess prosthesis survival.

### Statistical Analysis

All analyses were performed in SPSS 26.0 (IBM, Armonk, NY, USA). Normally distributed data were expressed as mean ± standard deviation (SD), otherwise data were expressed as median (25th percentile, 75th percentile). Continuous data such as age, follow‐up duration, HKA, were compared between knees with neutral or mild varus alignment using analysis of Student's *t* test or variance or the Kruskal–Wallis test as appropriate. For multiple comparisons of our primary outcome (ROM, WOMAC, KSS, KFS, and FJS), we used Bonferroni–Dunn method to adjust *p*‐values. Categorical data were compared using the *χ* ^2^ test. Differences associated with *p* < 0.05 and adjust *p* < 0.01 were considered significant.

## Results

### Demographic Data and Alignment among Two Groups

Of the 156 knees in 137 obese patients who completed follow‐up, 97 knees were corrected from varus to neutral, while 54 remained in mild varus. Age, sex, body mass index, preoperative HKA, and follow‐up duration did not differ significantly between patients whose knees had neutral alignment and those whose knees had mild varus (Table 1). Neutral knees were corrected from a preoperative mean of −8.7° to −1.0° (Table 2). Mild varus knees were corrected from a preoperative mean of −9.8° to −4.3°.

**TABLE 1:** Characteristics of patients stratified according to whether their knees showed neutral or mild residual varus alignment after total knee arthroplasty.

Characteristic	Neutral (*n* = 97 knees)	Mild varus (*n* = 54 knees)	*p* *
Patients	86	46	
Sex			0.174
Male	13	5	
Female	73	41	
Age (years)	72.84 (6.664)	74.39 (6.700)	0.921
Body mass index (kg/m^2^)	31.49 (1.355)	31.47 (1.441)	0.964
Preoperative HKA	−8.7 (4.962)	−9.8 (4.374)	0.255
Follow‐up (years)	8.13 (1.455)	8.62 (1.538)	0.060

*Note*: Values are *n* or mean (SD) unless otherwise noted

Abbreviations: HKA, hip‐knee‐ankle axis angle; TKA: total knee arthroplasty

* Based on Student's *t* test or Mann–Whitney test (continuous variables) or *χ* ^2^ test (categorical variables).

**TABLE 2:** Hip‐knee‐ankle axis angle before and after total knee arthroplasty (TKA) for knees stratified based on postoperative alignment.

Alignment	*n* (knees)	Preoperative	Postoperative
Neutral	97	−8.7 (4.962)	−1.0 (0.129)
Mild varus	54	−9.8 (4.374)	−4.3 (0.127)
Severe varus	1	−16.4	−7.3
Valgus	4	−3.51 (3.025)	3.84 (0.336)

*Note*: Values are *n* or mean (standard deviation).

### Postoperative Complications and Prosthesis Survival

One patient with a neutral knee had to undergo revision surgery due to aseptic loosening of the tibial component. *χ* ^2^ test showed no significant difference between the two groups (*p* > 0.999). At the last follow‐up or the last X‐ray examination, there was no prosthesis loosening or polyethylene liner wear in the other patients (Table 3). The Kaplan–Meier survival curve shows the survival of the prosthesis in the two groups (Figure 4). We further performed Log Rank survival analysis and showed no significant difference between the two groups (*p* = 0.425).

**TABLE 3:** Functional outcomes, postoperative complications, and prosthesis survival after TKA for knees stratified by postoperative alignment.

Score or outcome	Neutral (*n* = 97 knees)	Mild varus (*n* = 54 knees)	Adjust *P* *
Range of motion (°)	112.34 (17.320)	120.00 (13.633)	0.029
WOMAC score	14.97 (14.193)	8.25 (8.637)	0.009
Knee Society knee score	83.66 (10.640)	87.25 (8.095)	0.164
Knee Society function score	75.69 (20.139)	78.31 (17.366)	>0.999
Forgotten joint score	70.22 (30.031)	86.03 (15.607)	0.002
Polyethylene wearing	1	0	>0.999
Prosthesis loosening	1	0	>0.999
Revision	1	0	>0.999

*Note*: Values are *n* or mean (standard deviation) unless otherwise noted

Abbreviations: TKA, Total knee arthroplasty; WOMAC: Western Ontario and McMaster Universities Osteoarthritis Index score

* Bonferroni–Dunn method was used to adjust *p* value.

### Functional Outcomes

**FIGURE 3:**
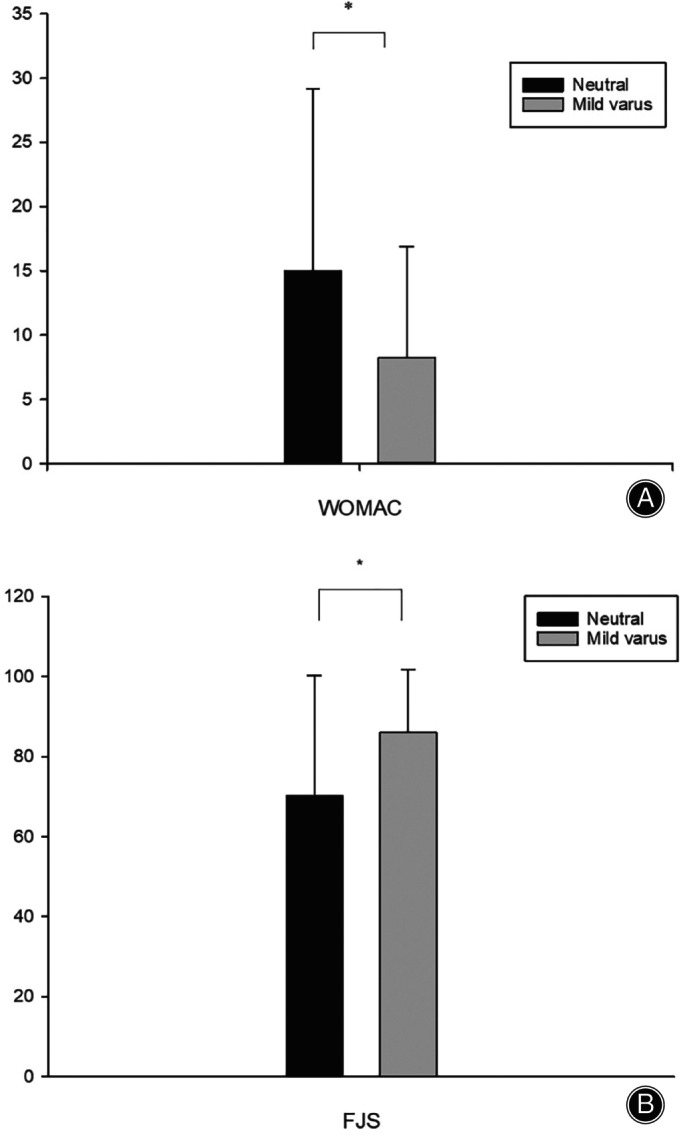
Differences in WOMAC (A) and FJS (B) between patients with neutral and mild varus. *p < 0.05. WOMAC, Western Ontario and McMaster Universities Osteoarthritis Index score. FJS, Forgotten Joint Score.

Patients whose knees had neutral or mild varus did not differ significantly in postoperative ROM, KS‐KS, or KS‐FS (Table 3). Patients with mild varus knees had significantly WOMAC (8.25 ± 8.637 vs. 14.97 ± 14.193, *p* = 0.009; Figure 3A) and better FJS (86.03 ± 15.607 vs. 70.22 ± 30.031, *p* = 0.002; Figure 3B).

**FIGURE 4:**
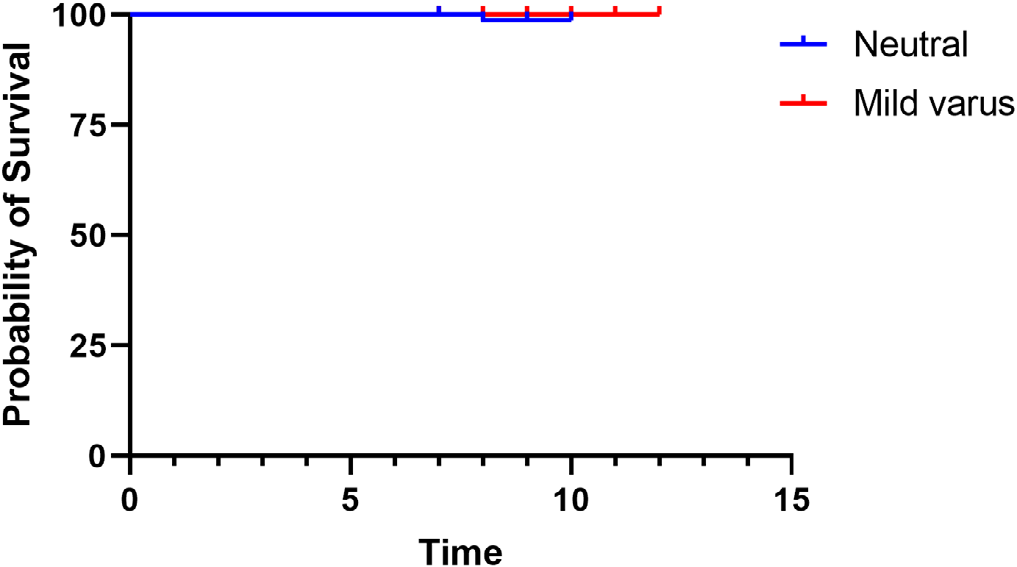
The Kaplan–Meier survival curve shows the survival of the prosthesis in patients with neutral and mild varus.

## Discussion

Our retrospective study suggests that there is no significant difference in prosthesis survival between obese patients who have neutral or mild residual varus after TKA. Nevertheless, we found evidence that mild postoperative varus alignment is associated with better outcomes than neutral alignment. Traditionally, optimal outcomes in TKA have been thought to depend on achieving a lower‐extremity HKA within 3° of neutral and positioning tibial and femoral components perpendicular to the mechanical axis in the coronal plane.^17^, ^18^ On the other hand, several studies^19^, ^20^ have questioned this long‐held tenet, arguing that it creates an unnatural situation for at least a subset of patients. Assessing the validity of this practice may be particularly important for obese patients, whose knees are overloaded and in whom the load is distributed onto the tibial component.^21^ Previous studies^10^, ^19^, ^22^, ^23^, ^24^, ^25^, ^26^, ^27^, ^28^, ^29^, ^30^ comparing outcomes between TKA patients with postoperative neutral or mild varus did not distinguish between obese and non‐obese patients.

### Postoperative Complications and Prosthesis Survival

Our finding that postoperative neutral or mild varus did not significantly affect prosthesis survival for obese patients after TKA. Similar results were found from several studies^22^, ^23^, ^24^, ^25^ that did not distinguish obese from non‐obese patients. However, it contrasts with the conclusion of a meta‐analysis^7^ of 10 studies involving 12,278 knees, according to which mild or severe residual varus shortens prosthesis survival. Instead, those investigators concluded that neutral or valgus alignment is essential for long‐term prosthesis survival. It is difficult to compare that meta‐analysis with our study because they did not distinguish between mild and severe varus. Our results also contrast with a study^26^ in which left 3° varus alignment was linked to adduction overload of the medial compartment, causing asymmetrical polyethylene liner wear, tibial component loosening, and mechanical failure. Our finding should be verified and extended in studies with medium to long follow‐up, given the length of time needed to adequately assess outcomes; and in studies involving morbidly obese patients, who were rare in our sample. Such patients may be at even higher risk of asymmetric polyethylene lining wear, loosening of tibial components, and mechanical failure.^2^

### Functional Outcomes

Our study suggests that mild postoperative varus alignment led to better functional outcomes than neutral alignment. Our results are similar to those of three studies^22^, ^29^, ^30^ reporting better outcomes for mild varus alignment after mean follow‐up of 4–7 years, but our results contrast with those of four studies^10^, ^19^, ^27^, ^28^ that failed to find a significant difference in outcomes for mean follow‐up for up to 5 years. One study^31^ has suggested that mild residual varus alignment can create physiological tension in the soft tissue that feels more “natural” to patients, and we speculate that this benefit may increase with time, which may explain why mainly studies with longer follow‐up have detected it. Our finding may be especially important not only because of our medium follow‐up but also because our assessment included the FJS, which measures patients' ability to forget the artificial joint in everyday life. This index is likely to be strongly associated with patient satisfaction.^32^ In recent years, kinematically aligned (KA) TKA was developed to restore normal knee function by maintaining the soft tissue envelope and minimizing the need for ligament release.^33^ However, mechanical alignment (MA) TKA may demand higher release of soft tissues and ligaments, especially in patients with severe varus, which contrasts with KA TKA concepts. Our results that it is not that important to merely generate a neutral HKA angle, lending support to the emerging view that TKA patients can benefit more from KA TKA than from MA TKA.^34^, ^35^, ^36^

### Strength and Limitations

The strength of this study is the grouping of obese patients with different joint types after TKA and the first establishment of an association between mild varus and better joint function after TKA in obese patients. These findings further expand the selection of preoperative and intraoperative alignment types. Nevertheless, our findings should be interpreted with caution in light of several limitations. First, our finding of no significant difference in prosthesis survival should be considered preliminary until it can be verified in larger samples with longer follow‐up. Second, we included only patients with primary knee osteoarthritis, which led to the same prominent female bias in our sample as in most other studies similar to ours.^37^ Third, we did not closely examine patients with severe preoperative varus, who require further study because soft tissue release is more challenging in such cases. Similarly, further study is needed that differentiates obese patients by the severity of their obesity and comorbidities. Finally, because the procedures were performed by the same surgeon, there is selection bias that may prevent our results from explaining the results of a wider range of procedures.

## Conclusion

Our retrospective study suggests that retaining slight varus in the knees of obese patients undergoing TKA may lead to better functional outcomes without reducing prosthesis survival, even in the medium term.

## Conflicts of Interest Statement

The authors have no relevant financial or non‐financial interests to disclose.

## Ethics Statement

Statement of informed consent: Informed consent was obtained from all individual participants included in the study. Statement of human animal rights: The study was approved by our institution research ethics committee.

## Author Contribution

All authors had full access to the data of the study and take responsibility for the integrity and interpretation of the data. Conceptualization: YH, Lai; ZK, Zhou. Methodology, YH, Lai; WX, Zhao; XY, Li. Investigation: YH, Lai; WX, Zhao; XY, Li; N, LV. Formal analysis: YH, Lai; WX, Zhao; XY, Li; N, LV. Resources: ZK, Zhou. Writing—original draft: YH, Lai. Writing—review and editing: YH, Lai; WX, Zhao; XY, Li. Visualization: ZK, Zhou. Supervision: ZK, Zhou.
